# A clinically actionable nomogram integrating HbA1c, renal function, and blood pressure for early prediction of diabetic macular edema in working-age patients with type 2 diabetes

**DOI:** 10.3389/fendo.2026.1710040

**Published:** 2026-02-25

**Authors:** Qingchun Pan, Lei Wang, Renli Huang, Xingya Li, Bei Li

**Affiliations:** 1 Affiliated Hospital of North Sichuan Medical College, Nanchong, China; 2 Longchang People's Hospital, Sichuan, Longchang, China

**Keywords:** eGFR, HbA1c, nomogram, systolic blood pressure, type 2 diabetes mellitus

## Abstract

**Objective:**

This study aims to identify factors associated with diabetic macular edema (DME) presence in working-age (18–60 years) patients with type 2 diabetes mellitus (T2DM) by developing a model that integrates HbA1c, renal function (eGFR), and hemodynamic parameters (SBP). The model addresses critical gaps in current screening strategies by using routinely available biomarkers, thereby enabling non-ophthalmologists to efficiently identify high-risk individuals.

**Methods:**

This cross-sectional study prospectively collected data from 490 patients with type 2 diabetes mellitus (T2DM), aged 18–60 years, who were consecutively enrolled at a single medical center between January 2020 and March 2025. The participants were randomly allocated into two groups: a training cohort (n=343) and a validation cohort (n=147). Predictors were selected via LASSO regression with 10-fold cross-validation from an initial set of 19 variables, encompassing renal function, metabolic parameters, and hemodynamic indices. Subsequently, a multivariate logistic regression model was developed and illustrated through a nomogram. The model’s predictive accuracy was evaluated through receiver operating characteristic (ROC) curves (AUC), calibration curves, and decision curve analysis (DCA).

**Results:**

The overall prevalence of DME in the study cohort was 15.71% (77 of 490). Four predictors independently associated with DME were identified using LASSO regression, namely diabetes duration (OR = 1.460, 95% CI: 1.212–1.457), SBP (OR = 1.066, 95% CI: 1.037–1.095), eGFR (OR = 0.938, 95% CI: 0.916–0.961), and HbA1c (OR = 1.484, 95%CI: 1.189–1.852). The resulting nomogram exhibited robust discriminatory ability (training AUC = 0.905, 95% CI: 0.858–0.951; validation AUC = 0.884, 95% CI: 0.820–0.949) and strong calibration performance (Hosmer-Lemeshow test, P = 0.878). DCA further confirmed substantial clinical applicability within a threshold probability range from 2% to 100%, achieving a maximum net benefit of 0.14, thereby potentially preventing unnecessary intervention in 14 out of every 100 patients.

**Conclusion:**

This nomogram effectively integrates HbA1c, renal function, and hemodynamic parameters to identify key factors associated with diabetic macular edema (DME) risk in working-age patients with type 2 diabetes mellitus (T2DM), demonstrating high accuracy. By utilizing routine clinical measures, it facilitates implementation in primary care settings, offering the potential to reduce vision loss through timely referrals. Future multicenter studies are warranted to verify its generalizability and explore its integration with emerging biomarkers.

## 1 Introduction

Diabetic macular edema (DME) is the leading cause of vision impairment in individuals with type 2 diabetes mellitus (T2DM), with prevalence rates ranging from 1.4% to 12.8% (1–3). The global rise in T2DM incidence is expected to increase the number of DME patients to over 30 million by 2045, imposing a substantial economic burden on healthcare systems (1–3). Early diagnosis is crucial for risk stratification, timely intervention, and cost reduction. The working-age population (18–60 years) is particularly vulnerable to DME-related visual impairment, which can lead to central vision loss, distorted vision, and impaired color perception—severely affecting occupational function and quality of life (4, 5). For individuals in visually demanding professions (e.g., drivers, programmers), DME may result in job loss, compounding socioeconomic challenges. Although anti-VEGF therapy remains first-line treatment, poor response rates (30–40%) and high costs limit its effectiveness (6). Frequent injections and follow-ups further reduce compliance among employed patients. Low screening rates in this age group often delay diagnosis, increasing the risk of irreversible vision loss. Thus, a reliable risk prediction tool is urgently needed for early identification of high-risk individuals.

Current screening relies heavily on fundus examination and optical coherence tomography (OCT). However, OCT is seldom available in primary care settings (7). Additionally, a management gap exists between internists overseeing diabetes care and ophthalmologists managing DME, complicating coordinated care. These challenges underscore the need for an accessible, efficient screening tool suitable for non-specialist use. Predictive models offer a promising solution by enhancing screening accessibility, enabling continuous monitoring, and optimizing resource allocation. Yet, no consensus exists on DME risk factors in working-age T2DM patients. The pathophysiology of DME involves chronic hyperglycemia, blood-retinal barrier disruption, inflammation, and oxidative stress (8). While American Diabetes Association (ADA) guidelines emphasize HbA1c and diabetes duration as key predictors, they overlook other metabolic and systemic factors (9). Other studies suggest roles for hypertension, dyslipidemia, nephropathy, and anemia (10). Previous prediction models have been limited by small samples, incomplete variable selection, and poor interpretability.

To address these limitations, we employed least absolute shrinkage and selection operator (LASSO) regression—a robust method for feature selection in chronic disease prediction (11). In this single-center cross-sectional study, we integrated metabolic, renal, and hemodynamic parameters to develop a clinically actionable nomogram for DME risk assessment in working-age T2DM patients. Our objectives were: (1) to establish a non-invasive risk tool for this population; (2) to evaluate the combined predictive value of routine biomarkers; and (3) to provide a visual, interpretable model for individualized intervention. This study aims to improve early detection, reduce unnecessary referrals, and ultimately mitigate vision loss in a high-risk, economically active demographic.

## 2 Methods

### 2.1 Research subjects

This cross-sectional study adopted a prospective data collection approach. We consecutively enrolled patients with type 2 diabetes mellitus (T2DM) aged 18–60 years who visited the hospital between January 2020 and March 2025.The cross-sectional design allows for examination of associations but does not permit inference of temporal relationships. Inclusion criteria were as follows (12): (1) meeting the 2018 diagnostic criteria of the American Diabetes Association; (2) aged 18–60 years; (3) completed standardized ophthalmic examinations; (4) complete clinical data. Exclusion criteria included: (1) other retinal diseases; (2) history of vitrectomy or anti-VEGF therapy; (3) malignant tumors or hematological diseases.

**Figure 1:**
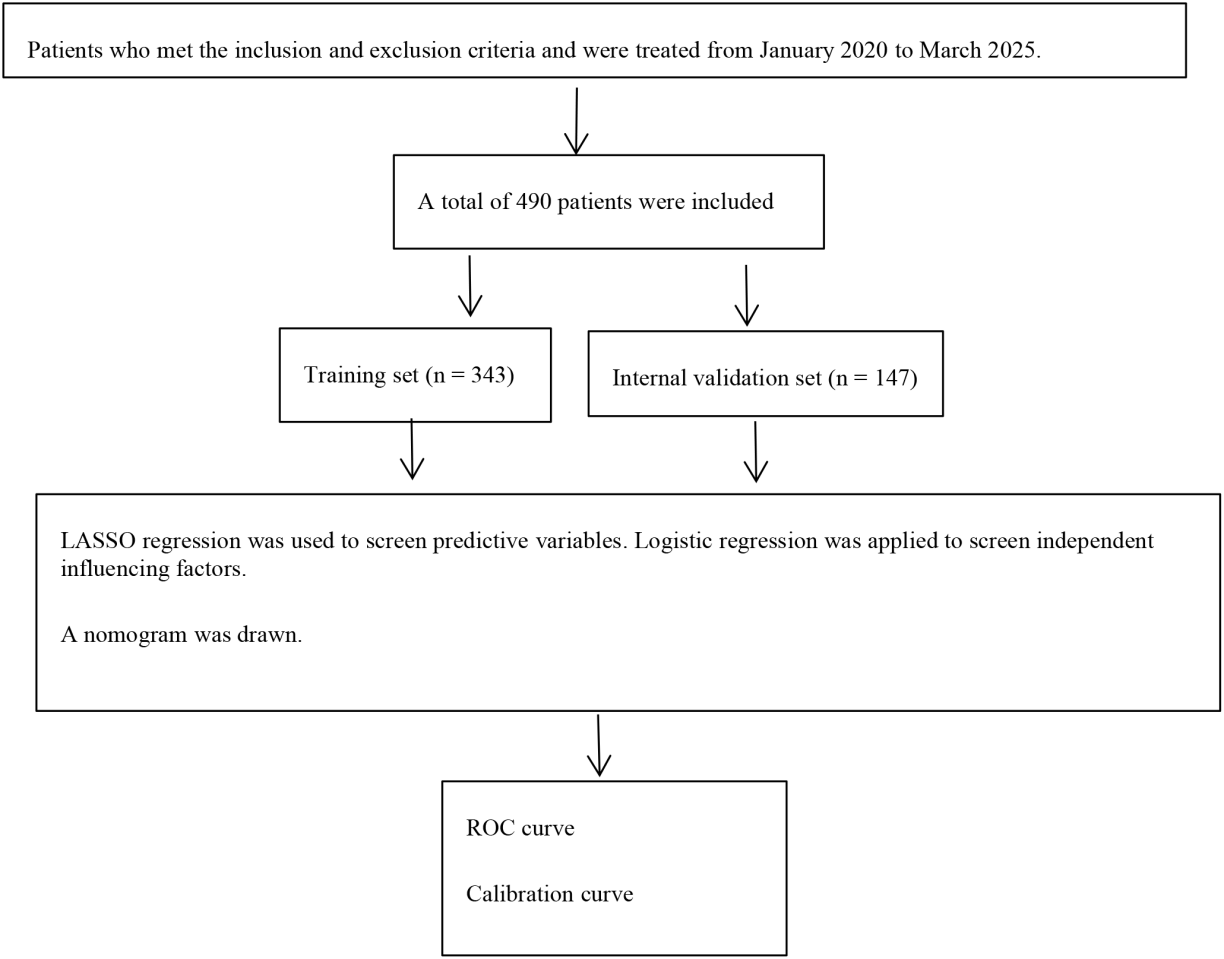
Research flowchart.

The sample size was calculated using a logistic regression model. This model required that the number of outcome events exceed ten times the number of independent variables. According to reporting guidelines for predictive models and risk-of-bias assessment tools, at least 10 independent variables were necessary (13). In this study, occurrence of DME served as the dependent variable. Based on the literature, 15 independent variables were anticipated in the predictive model, and the prevalence of DME among T2DM patients was approximately 40%. Therefore, the minimum sample size required was 375 (15×10÷40%). To account for potential invalid or incomplete samples, an additional 20% of participants were included, yielding a total enrollment of 490 patients. These participants were randomly allocated at a 7:3 ratio into the training (n=343) and validation cohorts (n=147). The methodological flowchart of the study is detailed in **Figure 1**. This study was approved by the ethics department of our hospital (Ethics Number: 2020ER035-1), and all subjects gave informed consent to the trial content.

### 2.2 Data collection

#### 2.2.1 Basic information

Data collection procedures were standardized across the study. Participant age information was verified using official identification documents. BMI values were determined using calibrated electronic scales for height (accurate to 0.1 cm) and weight (accurate to 0.1 kg), with BMI calculated as weight (kg)/[height (m)]^2^ (14).

#### 2.2.2 Selection of instrumental variables

Fundus examinations were conducted after mydriasis using a Topcon TRC-50DX fundus camera. Seven-field color fundus photographs were captured, including images of the macular center, four quadrants, optic disc, and wide-angle posterior pole. Two ophthalmologists (associate chief physician or higher) independently evaluated images using a double-blind method. Diabetic retinopathy was graded according to the ETDRS standard.

OCT examination was performed using Heidelberg Spectralis OCT and the ETDRS 9-sector grid scanning protocol. The mean thickness of the central 1-mm diameter area was automatically measured as the central subfield thickness (CST). Diagnostic thresholds were: normal (<250 μm), mild edema (250–300 μm), moderate edema (300–400 μm), and severe edema (≥400 μm) (15). Subretinal fluid was defined as a separation between the neuroepithelial layer and the RPE of ≥50 μm, and typical OCT features were recorded if present.

Diagnostic criteria for DME were: OCT-confirmed macular thickening (CST ≥250 μm) accompanied by at least one of the following: (1) low-reflective cystic cavities between retinal layers; (2) dark sub-neuroepithelial fluid spaces; or (3) hard exudate deposits (16).

#### 2.2.3 Clinical indicators

##### 2.2.3.1 Lifestyle and history data

Smoking history, alcohol consumption, and family history of diabetes were collected through standardized questionnaires. Diabetes duration was precisely calculated from the month of first meeting WHO (1999) diabetes diagnostic criteria.

##### 2.2.3.2 Blood pressure measurement

Blood pressure was measured using a validated electronic upper-arm sphygmomanometer after subjects sat quietly for 5 minutes. The average of three consecutive measurements was recorded.

##### 2.2.3.3 Laboratory analyses

HbA1c: For HbA1c analysis, approximately 2–3 mL of venous blood was drawn from the antecubital vein into EDTA-containing tubes. Participants were instructed to avoid strenuous physical activities and dietary extremes prior to blood sampling. Samples were then centrifuged to obtain plasma or serum, and HbA1c levels were subsequently quantified by high-performance liquid chromatography (HPLC), which differentiates glycated from non-glycated hemoglobin based on differences in ionic charge (17).

Fasting plasma glucose (FPG): Subjects fasted for 8–10 hours overnight. Venous blood (2–3 mL) was drawn between 7 and 9 a.m. from the median cubital vein after iodophor disinfection, using vacuum tubes. Samples were centrifuged within 2 hours to separate serum/plasma. Concentrations were determined using the glucose oxidase (GOD-POD) method, which involves enzymatic generation of peroxide quantified colorimetrically (18).

ALT and AST: Subjects fasted 8–12 hours, avoided alcohol, vigorous exercise, and high-fat foods. Venous blood (2–3 mL) was collected without anticoagulants or coagulants, gently inverted 5–8 times, and centrifuged within 2 hours (3,000 rpm, 10 min) to separate serum. Concentrations were measured by the rate method (19).

Estimated glomerular filtration rate (eGFR): Subjects avoided vigorous exercise and high-protein diets for 3 days before sampling. Venous blood (3–5 mL) was collected without anticoagulants, gently inverted 5–8 times, and centrifuged (3,000 rpm, 10 min) within 2 hours to separate serum. Serum creatinine was determined enzymatically (creatinine oxidase method). eGFR was calculated using the Modification of Diet in Renal Disease (MDRD) equation (20).

TC, LDL, and TG: Subjects fasted for 8–12 hours and maintained normal diets for 3 days, avoiding high-fat foods, alcohol, and vigorous exercise. Participants sat quietly for 5 minutes before sampling. Venous blood (3–5 mL) was collected without anticoagulants and centrifuged (3,000 rpm, 10 min) within 30–45 min to separate serum. Concentrations were measured by the cholesterol esterase method (CEH-CHOD-PAP) (21).

TC, LDL, and TG:: Subjects fasted for 12 hours, avoided alcohol and vigorous exercise, and sat quietly for 5 minutes before sampling. Venous blood (2–3 mL) was collected without anticoagulants or using vacuum tubes with separation gel. Samples were centrifuged (4,000 rpm, 10 min) within 30 min to separate serum. Concentrations were measured using the bromocresol green (BCG) method (22).

#### 2.2.4 Quality control system

A three-level quality control system was established for all test indicators. Laboratory indicators were evaluated daily using two levels of quality control materials and regularly participated in CAP external quality assessments (required score ≥90%). Questionnaires underwent double-entry with independent verification, including automatic logical checks. Clinical parameters were extracted automatically from electronic medical records. Key indicators were cross-verified against initial diagnosis records. Additionally, a three-level (red-orange-yellow) early warning system was implemented. A yellow warning (three abnormal indicators) required increased follow-up; an orange warning (five abnormal indicators) indicated the need for specialist consultation; a red warning (≥seven abnormal indicators) triggered immediate multidisciplinary intervention.

### 2.3 Statistical analysis

Statistical analysis was conducted using R software (version 4.2.0; R Foundation for Statistical Computing, Vienna, Austria). Predictors were identified by applying LASSO regression combined with 10-fold cross-validation, with the optimal λ parameter selected according to the minimum mean square error (MSE). This statistical method effectively reduces coefficients of insignificant variables to zero, thereby improving the accuracy and efficiency of prediction. Variables deemed significant by LASSO regression subsequently entered a multivariate logistic regression analysis, from which odds ratios (OR) and corresponding 95% confidence intervals (95% CI) were determined. A nomogram was then developed utilizing the regression coefficients derived from this model.

The validity and clinical applicability of the model were assessed from three distinct perspectives: (1) Discriminative capacity, evaluated through receiver operating characteristic (ROC) curve analysis, with the area under the ROC curve (AUC) and its associated 95% CI computed; (2) Calibration accuracy, assessed by plotting calibration curves and employing the Hosmer–Lemeshow test to quantify consistency between observed and predicted outcomes; (3) Clinical utility, estimated by decision curve analysis (DCA), which quantified the model’s net benefit across varying threshold probabilities to determine the optimal threshold for intervention.

Continuous variables were summarized as mean ± standard deviation (SD) if normally distributed, or as median and interquartile range [M (P25, P75)] if non-normally distributed. Categorical variables were presented as frequencies with corresponding percentages. Statistical significance was defined as a two-tailed P-value <0.05.

## 3 Results

### 3.1 Comparison of clinical data between training and validation sets

In total, 490 T2DM patients participated in the study, with 77 (15.71%) diagnosed with DME. Participants were randomly allocated to either a training cohort (n=343; DME prevalence: 15.74%, 54/343) or a validation cohort (n=147; DME prevalence: 15.65%, 23/147). Comparative analysis revealed no statistically significant differences between the two cohorts in terms of baseline clinical parameters (P>0.05, **Table 1**).

**Table 1:** Baseline characteristics of patients in the training and internal validation sets.

	Total (n=490)		Training set (n=343)		Internal validation set (n=147)		*X^2^/F/H*	*P*
	Non-DME (n=417)	DME (n=73)	Non-DME (n=289)	DME (n=54)	Non-DME (n=124)	DME (n=23)		
Gender (n,%)							1.801	0.180
Male	220 (53.27)	36 (46.75)	154 (53.29)	32 (59.26)	66 (53.23)	4 (17.39)		
Female	193 (46.73)	41 (53.25)	135 (46.71)	22 (40.74)	58 (46.77)	19 (82.61)		
Age (years)	51.06 ± 11.52	55.18 ± 10.6	50.77 ± 11.76	54.85 ± 11.05	51.73 ± 10.97	55.96 ± 9.66	-0.861	0.390
BMI (x ± s,kg/m2 )	23.81 ± 2.09	23.90 ± 2.27	23.70 ± 2.08	23.91 ± 2.28	24.07 ± 2.11	23.87 ± 2.31	-1.498	0.135
A long history of smoking (n,%)							0.090	0.764
No	287 (69.49)	55 (71.43)	200 (69.2)	38 (70.37)	87 (70.16)	17 (73.91)		
Yes	126 (30.51)	22 (28.57)	89 (30.8)	16 (29.63)	37 (29.84)	6 (26.09)		
History of alcohol abuse (n,%)							0.469	0.494
No	350 (84.75)	65 (84.42)	245 (84.78)	48 (88.89)	105 (84.68)	17 (73.91)		
Yes	63 (15.25)	12 (15.58)	44 (15.22)	6 (11.11)	19 (15.32)	6 (26.09)		
Family history of DM (n,%)							0.159	0.69
No	351 (84.99)	71 (92.21)	245 (84.78)	49 (90.74)	106 (85.48)	22 (95.65)		
Yes	62 (15.01)	6 (7.79)	44 (15.22)	5 (9.26)	18 (14.52)	1 (4.35)		
DM disease course (years)	8.3 (7.1,9.6)	10.2 (8.4,12.3)	8.2 (7,9.6)	10.5 (8.4,12.3)	8.7 (7.5,9.85)	9.5 (7.1,12.9)	-1.088	0.277
SBP (mmHg)	124.64 ± 14.99	138.75 ± 15.42	124.11 ± 14.82	139.52 ± 16.49	125.86 ± 15.39	136.96 ± 12.7	-0.677	0.499
DBP (mmHg)	78.80 ± 7.86	82.06 ± 9.40	78.84 ± 7.84	81.76 ± 9.75	78.72 ± 7.96	82.78 ± 8.68	-0.066	0.947
HbA1c (%)	9.20 ± 2.05	11.01 ± 1.88	9.13 ± 2.01	10.96 ± 2.02	9.39 ± 2.13	11.14 ± 1.53	-1.183	0.238
FPG (mmol/L)	7.52 ± 1.49	8.21 ± 1.70	7.53 ± 1.43	8.16 ± 1.68	7.49 ± 1.63	8.31 ± 1.78	0.061	0.951
ALT (U/L)	30.3 (28.2,32.5)	30 (27.9,32.2)	30.2 (27,33.3)	29.6 (27.4,32.3)	30.5 (29.6,31.35)	30.3 (28.2,31.7)	-0.942	0.346
AST (U/L)	30.5 (27.8,32.5)	29.9 (26.3,33)	29.9 (26.5,33.3)	30.35 (26.3,33.6)	30.65 (29.65,31.5)	29.4 (26, 33)	-0.983	0.325
eGFR [ml/ (min·1.73 m2 )]	101.25 ± 19.01	79.47 ± 22.95	101.52 ± 18.99	78.98 ± 22.36	100.63 ± 19.13	80.63 ± 24.76	0.224	0.823
TC (mmol/L)	3.90 ± 1.43	4.61 ± 1.48	3.95 ± 1.46	4.59 ± 1.51	3.77 ± 1.38	4.66 ± 1.42	0.969	0.333
LDL (mmol/L)	2.47 ± 0.98	2.77 ± 0.99	2.43 ± 0.98	2.80 ± 0.94	2.57 ± 1.00	2.71 ± 1.13	-1.082	0.280
TG	1.51 ± 0.55	1.74 ± 0.44	1.52 ± 0.57	1.72 ± 0.47	1.49 ± 0.50	1.79 ± 0.37	0.230	0.818
ALB (g/L)	31.98 ± 5.30	32.31 ± 4.54	31.89 ± 5.16	32.23 ± 4.63	32.18 ± 5.64	32.52 ± 4.43	-0.556	0.579
TP (g/L)	64.49 ± 7.39	63.72 ± 6.15	64.40 ± 7.66	63.53 ± 6.25	64.70 ± 6.74	64.17 ± 6.02	-0.509	0.611

### 3.2 Univariate analysis of DME in T2DM patients in the training set

**Figure 2:**
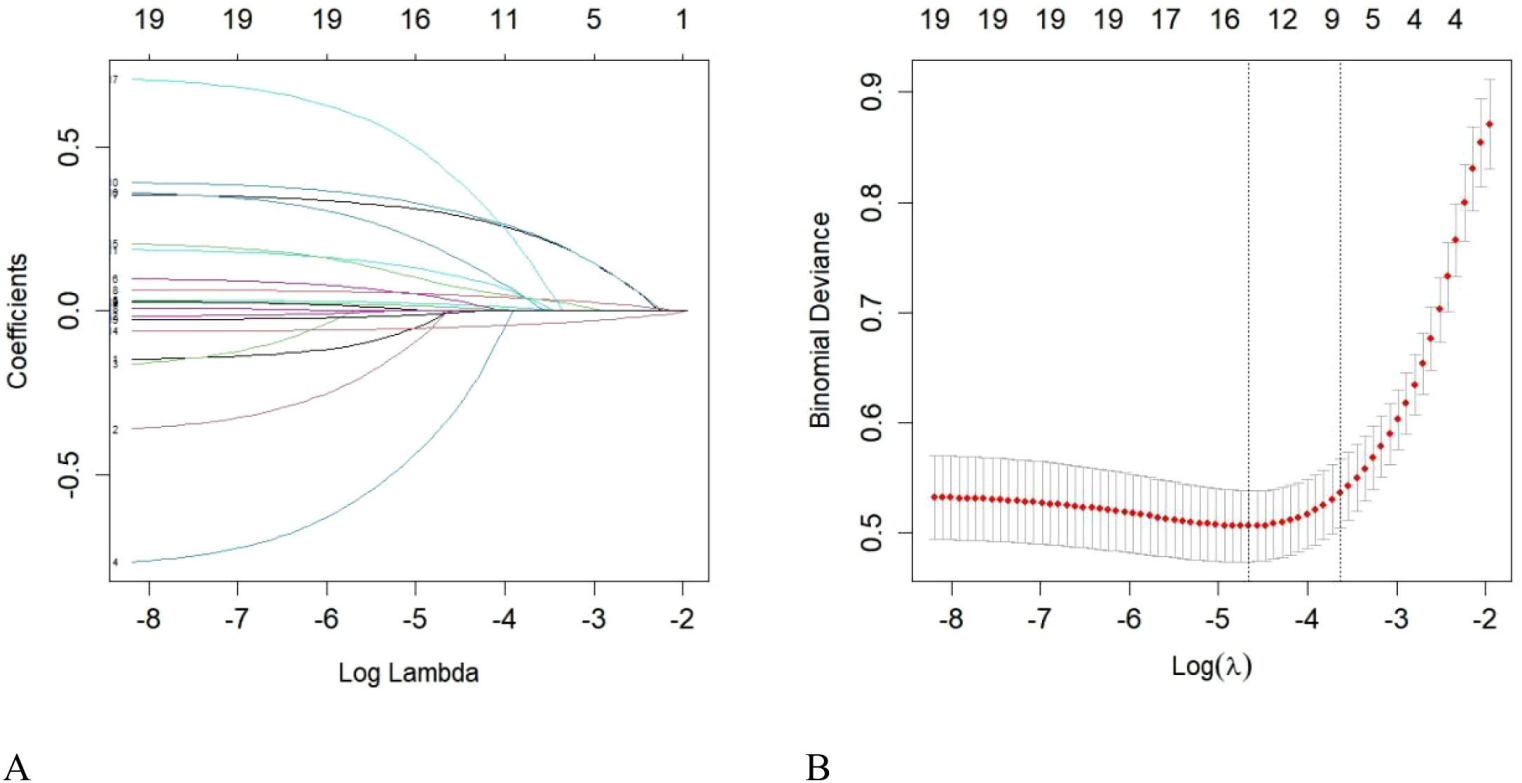
LASSO regression results (**A**: Variable selection; **B**: Cross-validation).

A total of 343 patients with T2DM were included in the training set. LASSO regression screened 19 candidate variables with non-zero coefficients, including gender, age, BMI, smoking, alcohol consumption, family history of DM, diabetes duration, SBP, DBP, ALT, AST, eGFR, FPG, HbA1c, TC, LDL-C, TG, ALB, and TP. Ten-fold cross-validation was conducted to determine the optimal λ parameter, reducing variables while maintaining model fit. After screening, nine predictors with non-zero coefficients remained: age, diabetes duration, SBP, eGFR, FPG, HbA1c, TC, LDL-C, and TG (**Figure 2**).

### 3.3 Logistic regression analysis of factors influencing DME in T2DM patients

Logistic regression analysis was performed using DME as the dependent variable. Independent variables evaluated included patient age, diabetes duration, SBP, eGFR, FPG, HbA1c, TC, LDL-C, and TG. Results indicated that diabetes duration, SBP, eGFR, and HbA1c were significantly associated with DME development (**Table 2**).

**Table 2:** Logistic regression analysis of factors influencing DME in T2DM patients.

	B	S.E.	Wald	P	OR	95%CI	
Age	0.035	0.021	2.824	0.093	1.035	0.994	1.078
DM disease course (years)	0.379	0.094	16.145	<0.001	1.460	1.214	1.757
SBP(mmHg)	0.064	0.014	20.84	<0.001	1.066	1.037	1.095
HbA1c	0.395	0.113	12.167	<0.001	1.484	1.189	1.852
FPG	0.199	0.142	1.980	0.159	1.221	0.925	1.611
eGFR	-0.064	0.012	26.719	<0.001	0.938	0.916	0.961
TC	0.168	0.150	1.251	0.263	1.183	0.881	1.589
LDL	0.362	0.214	2.861	0.091	1.436	0.944	2.183
TG	0.712	0.405	3.089	0.079	2.038	0.921	4.509
Quantity	-17.936	3.221	31.01	0.000	0.000		

### 3.4 Construction and evaluation of nomogram model for T2DM patients with DME

**Figure 3:**
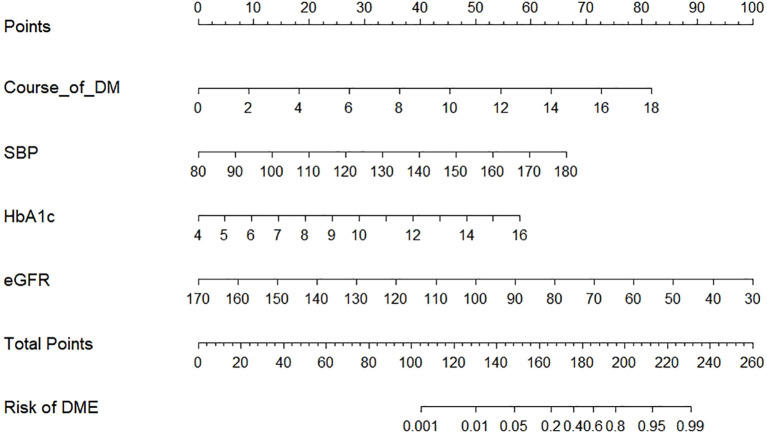
Nomogram model for DME in T2DM patients.

Based on multivariate logistic regression analysis, a nomogram predicting DME risk in T2DM patients was constructed using diabetes duration, SBP, eGFR, and HbA1c (**Figure 3**). This nomogram provided a visual tool for individualized risk assessment. The usage method involved locating each patient’s values on corresponding variable axes, summing scores on the “Total Score” axis, and projecting the total onto the “Risk” axis to determine predicted probability.

### 3.5 Model validation

**Figure 4:**
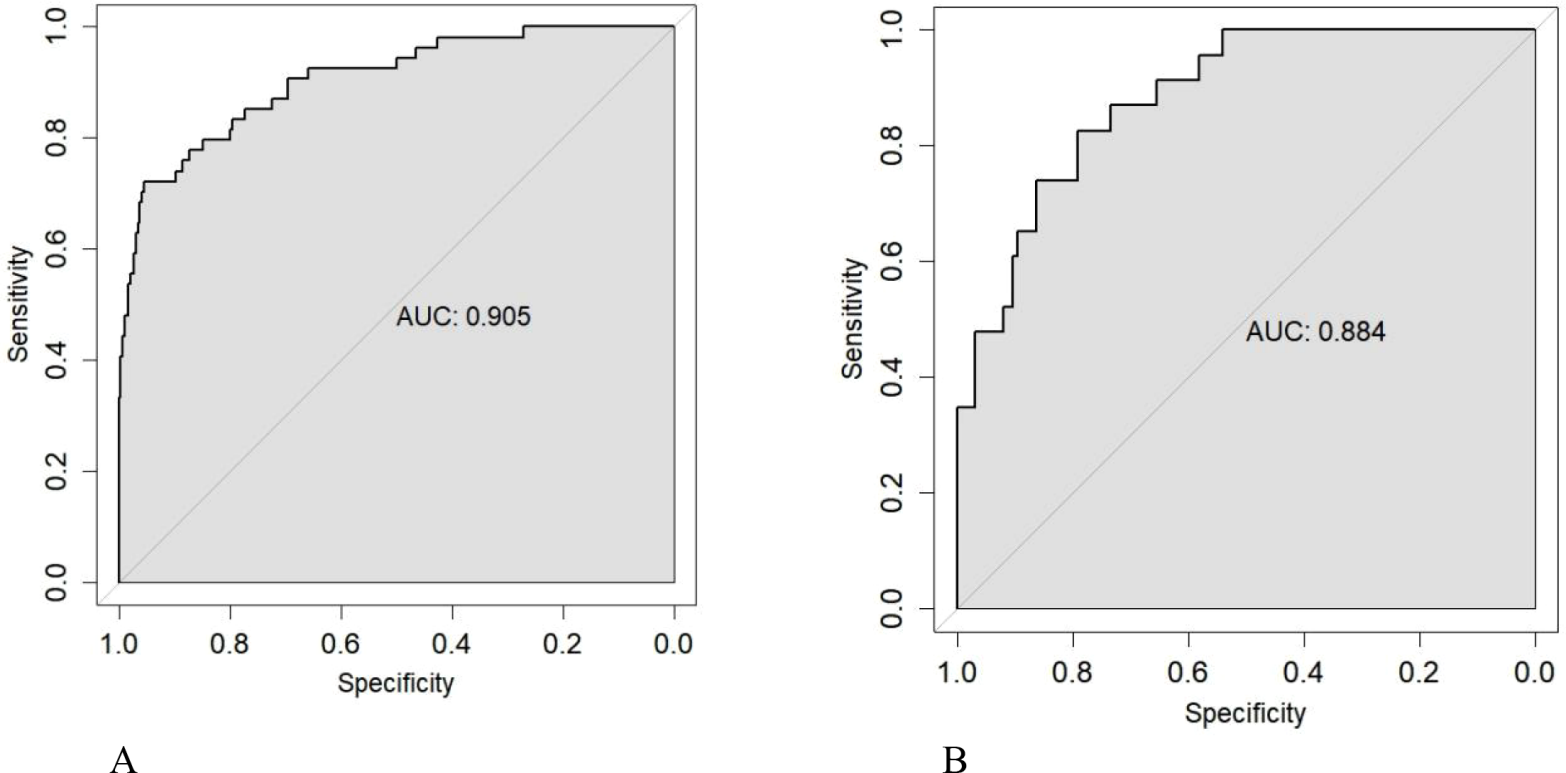
ROC curves of the model in training **(A)** and internal validation sets **(B)**.

The four variables identified from logistic regression were included in the nomogram (**Figure 3**). ROC curve analysis was conducted to evaluate model discrimination. Results showed excellent discrimination between T2DM patients with and without DME. The training set AUC was 0.905 (95% CI: 0.858–0.951; **Figure 4A**), and the validation set AUC was 0.884 (95% CI: 0.820–0.949; **Figure 4B**), outperforming individual variables.

**Figure 5:**
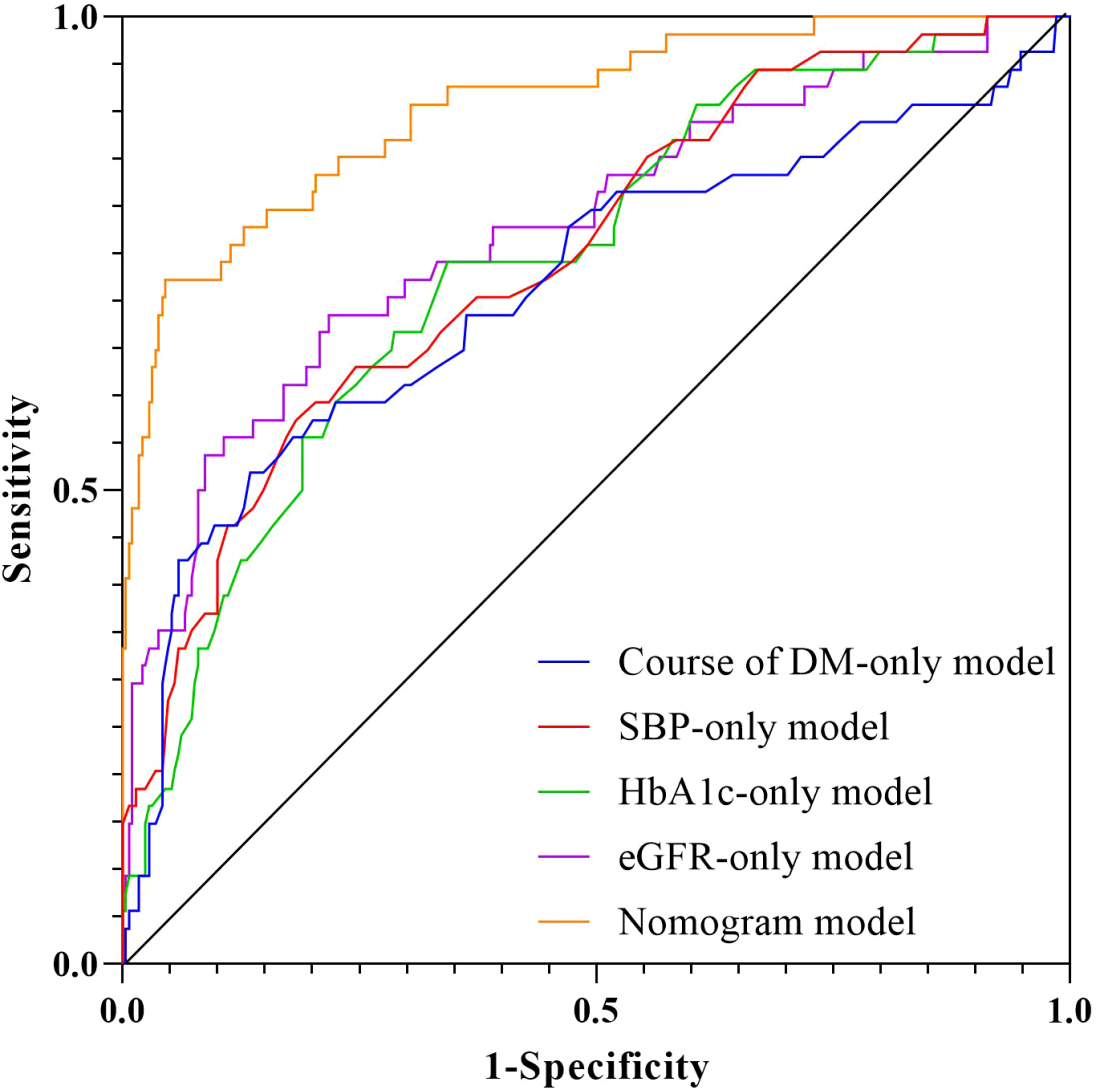
ROC curve analysis of individual factors.

A systematic comparison was performed between single-variable models and the comprehensive nomogram. Delong test results showed statistically superior predictive ability of the comprehensive model compared to single-variable models (**Figure 5**, **Table 3**).

**Table 3:** Comparison of individual factors and nomogram predictive values.

	AUC	95%CI	P	Sensitivity	Specificity	Cut-off	Delong Z	P
Course of DM-only model	0.713	0.627-0.799	<0.001	51.9	86.5	10.45	4.452	<0.001
SBP-only model	0.748	0.676-0.821	<0.001	57.4	81.7	137.5	4.263	<0.001
HbA1c-only model	0.740	0.669-0.812	<0.001	74.1	65.7	9.95	4.261	<0.001
eGFR-only model	0.777	0.703-0.851	<0.001	68.5	78.2	-86.5	3.805	<0.001
Nomogram model	0.905	0.858-0.951	<0.001	72.2	95.5			

Delong test results indicated that predictions from the nomogram were significantly different from those of the other four models.

**Figure 6:**
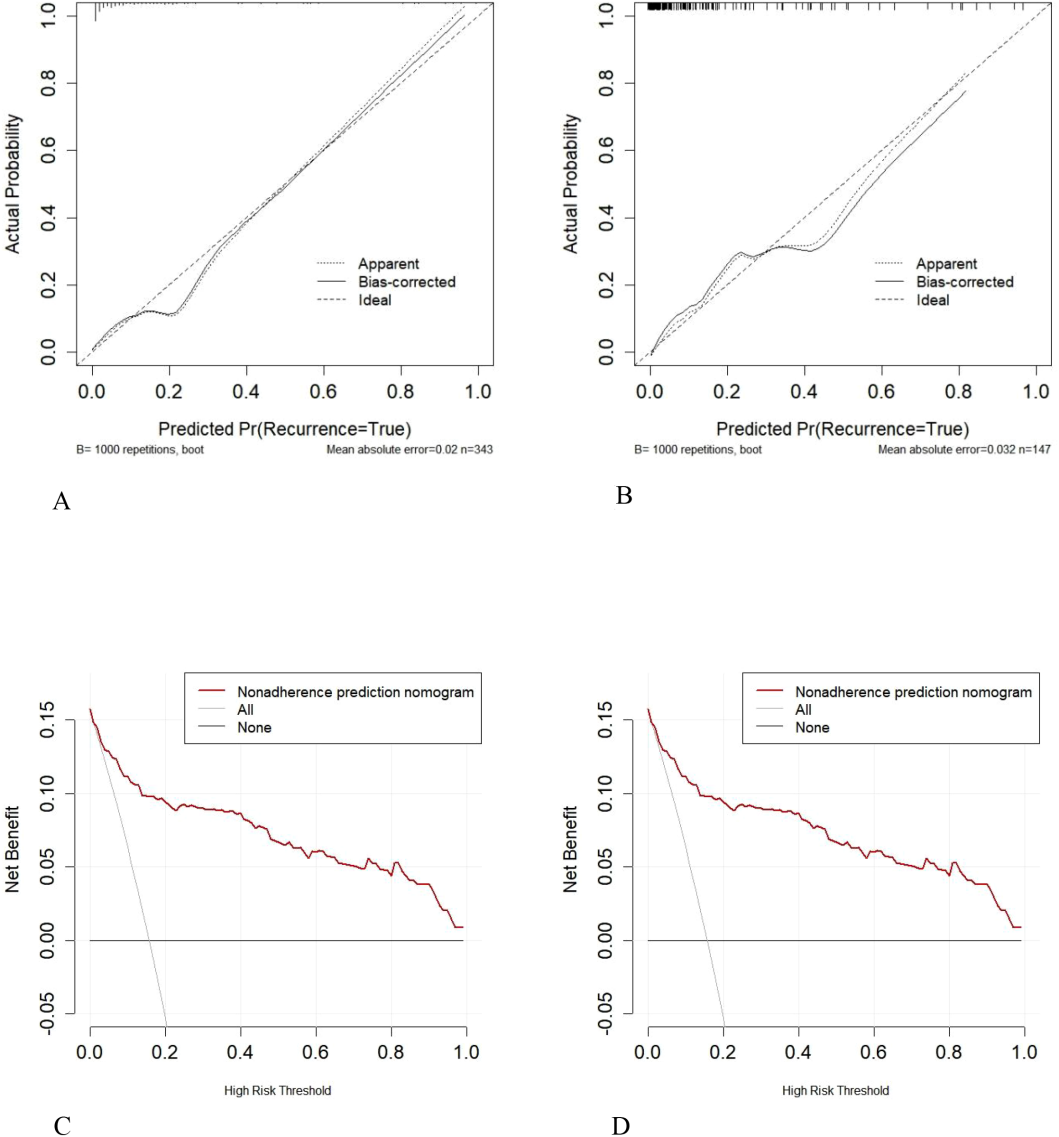
Calibration curves **(A, B)** and decision curves **(C, D)** for the training and internal validation sets.

Calibration curve analysis showed high consistency between predicted and actual probabilities (**Figures 6A, B**). The average calibration error was 0.020 (training set, Hosmer-Lemeshow test: χ²=3.748, P = 0.879) and 0.032 (validation set, Hosmer-Lemeshow test: χ²=5.024, P = 0.755). DCA evaluated net clinical benefit (**Figures 6C, D**). In the training cohort, the nomogram’s net benefit surpassed those of the “all-intervention” or “no-intervention” strategies across threshold probabilities ranging from 2% to 100%, achieving a peak net benefit of 0.14, implying the avoidance of 14 unnecessary interventions per 100 patients. Similarly, in the validation cohort, the net benefit remained consistently superior to the reference strategies at thresholds between 1.8% and 100%, supporting its effectiveness within moderate-to-high-risk groups.

## 4 Discussion

In this study, we developed and validated a nomogram for predicting diabetic macular edema (DME) risk specifically in working-age adults (18–60 years) with type 2 diabetes mellitus (T2DM). Using LASSO regression, we identified four key predictors—diabetes duration, systolic blood pressure (SBP), estimated glomerular filtration rate (eGFR), and HbA1c—and integrated them into a clinically actionable model. The nomogram demonstrated excellent discriminatory power, with AUC values of 0.905 and 0.884 in the training and validation sets, respectively, and well-calibrated prediction accuracy.

### 4.1 Novel contributions and clinical implications

This study addresses a critical gap in DME risk prediction by developing the first nomogram specifically tailored for working-age T2DM patients using exclusively routine clinical parameters. Unlike previous models that required specialized ophthalmologic equipment or focused on older populations, our tool leverages readily available biomarkers (HbA1c, eGFR, SBP) already collected in primary care settings. This approach makes DME risk assessment accessible to non-ophthalmologists, potentially transforming screening workflows in resource-limited environments. By enabling early identification of high-risk individuals in primary care, our model facilitates timely referrals to ophthalmology, optimizes resource allocation, and may significantly reduce vision loss in a demographic where visual impairment carries substantial socioeconomic consequences. The integration of renal function (eGFR) with metabolic and hemodynamic factors also provides new insights into the multifactorial pathophysiology of DME, highlighting the interconnectedness of microvascular complications in diabetes.

### 4.2 Interpretation of key predictors

The duration of diabetes emerged as a strong predictor (OR = 1.460), consistent with established literature linking longer disease duration to microvascular damage (23, 24). The slightly lower odds ratio compared to some previous studies may reflect better overall diabetes management in our younger cohort or their preserved metabolic compensatory mechanisms. Pathophysiologically, prolonged hyperglycemia drives DME through advanced glycation end-product accumulation, oxidative stress, and hemodynamic abnormalities that disrupt the blood-retinal barrier (25, 26). Our findings support intensified screening (e.g., every 3–6 months) for patients with diabetes duration ≥10 years.

Systolic blood pressure (SBP) was independently associated with DME risk (OR = 1.066), aligning with large-scale studies emphasizing blood pressure control in diabetic retinopathy (27). Notably, diastolic pressure showed no significant association, suggesting pulse pressure may be more relevant in DME pathogenesis. Hypertension likely contributes to DME by damaging endothelial tight junctions and promoting VEGF expression (28, 29).

The inverse relationship between eGFR and DME risk (OR = 0.938) underscores the role of renal dysfunction in DME development. This association may be explained by uremic toxin accumulation, chronic inflammation, and renal anemia-induced retinal hypoxia (30–33). Our results support integrating renal function assessment into DME risk stratification.

HbA1c levels significantly predicted DME risk (OR = 1.484), consistent with the known role of chronic hyperglycemia in microvascular complications (34, 35). The persistence of elevated risk even in patients with currently controlled HbA1c highlights the “metabolic memory” phenomenon (35–38), emphasizing the importance of early and sustained glycemic control.

## 5 Limitations

While this study successfully developed an effective DME prediction model, several limitations warrant careful consideration. The single-center, cross-sectional design, despite employing prospective data collection, may limit the generalizability of the findings, particularly as the sample was drawn from a single institution and external validation was not performed. Furthermore, the observed DME prevalence (15.7%) differed substantially from the initial estimate used for sample size calculation (40%), and the relatively small number of DME events compared to candidate predictors raises concerns about potential overfitting, despite the use of LASSO regression. It is also critical to acknowledge that the cross-sectional nature of the study means that temporal causality cannot be inferred from the observed associations. Clinically, the model’s scope is limited by the deliberate exclusion of specialized imaging parameters, such as OCT-measured retinal thickness, which are known to correlate with DME severity, and by not accounting for the impact of interventions like anti-VEGF therapy that can alter DME progression. Finally, while focusing on working-age adults (18–60 years) enhances relevance for that demographic, it restricts applicability to older populations who bear a higher disease burden.

These limitations highlight clear directions for future research. Priority should be given to conducting multi-center prospective studies with larger, more diverse cohorts to enable external validation and refine the model’s general applicability. Subsequent model iterations could significantly enhance predictive power by incorporating multi-modal imaging data, such as OCT and color fundus photography, and by considering the modifying effects of standard treatments. Further research should also explore optimal intervention thresholds and validate the model’s performance specifically in older age groups to provide more comprehensive clinical guidance.

## 6 Conclusion

This nomogram effectively integrates HbA1c, renal function, and hemodynamic parameters to identify key factors associated with diabetic macular edema (DME) risk in working-age patients with type 2 diabetes mellitus (T2DM), demonstrating high accuracy. By utilizing routine clinical measures, it facilitates implementation in primary care settings, offering the potential to reduce vision loss through timely referrals. Future multicenter studies are warranted to verify its generalizability and explore its integration with emerging biomarkers.

## Data Availability

The original contributions presented in the study are included in the article/supplementary material. Further inquiries can be directed to the corresponding author.
